# Huzhang Tongfeng Granule Improves Monosodium Urate-Induced Inflammation of Gouty Arthritis Rat Model by Downregulation of Cyr61 and Related Cytokines

**DOI:** 10.1155/2020/9238797

**Published:** 2020-04-28

**Authors:** Mi Zhou, Kan Ze, Yifei Wang, Xin Li, Liang Hua, Yi Lu, Xi Chen, Xiaojie Ding, Siting Chen, Yi Ru, Ming Zhang, Bin Li

**Affiliations:** ^1^Department of Dermatology, Yueyang Hospital of Integrated Traditional Chinese and Western Medicine, Shanghai University of Traditional Chinese Medicine, Shanghai 200437, China; ^2^Institute of Dermatology, Shanghai Academy of Traditional Chinese Medicine, Shanghai 201203, China

## Abstract

**Objective:**

Gouty arthritis (GA) is a noninfectious inflammatory disease characterized by self-limited and severe pain. Huzhang Tongfeng granule is one of the most effective traditional Chinese medicines in the treatment of acute GA. However, its effects on the inflammatory factors in the process of acute gout inflammation remain unknown. In the present study, we aimed to evaluate the effect of Huzhang Tongfeng granule on the expressions of Cyr61 and related inflammatory factors in both experimental gout models in vivo and in vitro.

**Methods:**

Huzhang Tongfeng granule was provided by the pharmaceutical preparation room of Yueyang Hospital of Integrated Traditional Chinese and Western Medicine. The expressions of Cyr61, IL-1*β*, TNF-*α*, and IL-6 in monosodium urate- (MSU-) induced rat models and fibroblast-like synoviocytes (FLSs) were determined by RT-PCR, Western blotting analysis, ELISA, immunohistochemistry, and hematoxylin and eosin staining.

**Results:**

Huzhang Tongfeng granule could downregulate the expressions of IL-1*β*, TNF-*α*, and IL-6 to some extent by inhibiting the expression of Cyr61.

**Conclusions:**

Collectively, our findings indicated that Cyr61 was highly expressed in rat models of gout. By inhibiting the expression of Cyr61, Huzhang Tongfeng granule could partially attenuate the inflammation induced by MSU crystal.

## 1. Introduction

Acute gouty arthritis (GA) is a group of clinical syndromes caused by monosodium urate (MSU) crystal deposition on bone, joints, and subcutaneous tissues, which is the most common initial symptom of gout [1]. Due to the change of population structure and diet composition, the incidence rate of acute GA in mainland China from 2000 to 2014 has been increased year by year, bringing pain to more and more patients [2]. Therefore, it is of great significance to explore the occurrence and development of GA for clinical use and relief of clinical symptoms.

The acute gout attack is an acute inflammatory process induced by MSU. When the solubility of uric acid in blood is too high, MSU is gradually deposited in the articular cartilage, synovium, and surrounding tissues. The precipitated MSU crystals act as foreign bodies to induce the chemotactic aggregation of leukocytes. They are also transformed into a signal that strongly stimulates the toll-like receptors (TLRs) and NOD-like receptor family pyrin domain-containing-3 (NLRP3) inflammasome, which activate the innate immune signaling pathway [3] and produce a series of inflammatory factors. IL-1*β* is the key inflammatory mediator to induce GA, and its production and release is the first and most important event in the process of gout inflammation. Studies have confirmed that monocytes and phagocytes in blood and synovial fluid of joints secrete IL-1*β* under the stimulation of MSU crystal and release a large amount of IL-1*β* out of the cells, causing acute inflammatory reaction [4]. MSU crystal can directly stimulate monocytes in synovial fluid of joint, resulting in TNF-*α* production on a large scale, which enhances the activity of neutrophils and leads to mass production and release of IL-1*β* [5]. It has been found that the degree of inflammatory activity in GA patients is associated with the IL-6 level, and thus IL-6 is considered to be related to the disease activity of gout [6].

Acute GA is characterized by spontaneous remission after the onset of inflammation, which is different from other arthropathy or spontaneous inflammatory diseases. With the progress of the disease, it gradually evolves into chronic GA. We have a certain understanding of the mechanism of inflammation in GA. However, the mechanism underlying the inflammation maintenance remains largely unexplored.

It has been found that cysteine-rich 61 (Cyr61) protein plays an important role in the maintenance of inflammation in arthritis. Cyr61 is an important extracellular matrix protein with proinflammatory function [7]. Normally, the expression of Cyr61 is low to maintain the physiological needs of the body, while many factors (such as inflammatory factors, growth factors, and mechanical tension stimulation) can lead to the increase of Cyr61 synthesis [8–10]. It has been found that Cyr61 is expressed in inflammatory diseases, such as arthritis and glomerulonephritis, and neuroinflammatory diseases, and its expression aggravates the inflammatory reaction [11]. Through *α*v*β*5/Akt/NF-κB signaling pathway, Cyr61 can stimulate synovial cells to secrete high concentrations of IL-6, which promotes the differentiation and proliferation of local Th0 cells into Th17 and the production of IL-17, forming a new inflammatory pathway for rheumatoid arthritis (RA) local arthropathy [12]. Cyr61 can also promote the production of IL-8 with the synovial cells in patients with RA, which mediates the chemotaxis of neutrophils to the joints and aggravates inflammation [13]. Cyr61 can widely participate in the pathological damage of inflammation and autoimmune diseases, and it is an important factor in the maintenance of inflammation [13–16]. The mechanism of Cyr61 in gout inflammation has not been reported. An in-depth study of the relationship between Cyr61 and gout inflammation is of great significance for elucidating the pathogenesis of GA. Our latest study has shown that high expression of Cyr61 protein can induce synovial cells to produce a large number of inflammatory cytokines, which is partially dependent on NF-*κ*B pathway [17].

Traditional Chinese medicine (TCM) has a long history as a method to prevent and treat GA. Preliminary studies have found herbal formula treatment to be efficient in the control of GA. Our study has found that there is no significant difference between Huzhang Tongfeng granule and diclofenac sodium in controlling acute gout inflammation [18]. Huzhang Tongfeng granule can significantly decrease the number of leukocytes and polymorphonuclear leukocytes, erythrocyte sedimentation rate, C-reactive protein, IL-6, and other inflammatory indexes in the blood of patients with acute GA [19]. The mechanism of action may be that it interferes with the assembly or activation of the NLRP3 inflammatory complex by downregulating NLRP3 and caspase-1 proteins and then antagonizing the inflammatory response of gout [20].

Based on the above-mentioned evidence, the present study aimed to clarify whether Huzhang Tongfeng granule could inhibit the expressions of inflammatory factors by regulating the expression level of Cyr61, thus antagonizing gout inflammation.

## 2. Materials and Methods

### 2.1. Drug Preparation

Huzhang Tongfeng granule was composed of 12 Chinese herbs (Table 1). The dosage used in this study was determined according to the Chinese Pharmacopoeia (2015 edition), and Huzhang Tongfeng granule was made by the Pharmacy Department of Yueyang Hospital of Integrated Traditional Chinese and Western Medicine, Shanghai University of Traditional Chinese Medicine. Briefly, (1) the ingredients were placed in a multifunctional extraction tank, and eight and four volumes of water were added, respectively. The extraction was carried out twice, and the water extract was boiled at 85°C for 20 min, filtered and pooled. (2) The liquid was put into the concentration tank with a circulating pump, and concentrated to an appropriate amount. (3) The supernatant was filtered by 80-mesh stainless steel screen, stevioside and dextrin were added, and the mixture was stirred in a blender. (4) The dried powder was put into FC160C/B electric high-speed crusher to make powder. (5) The powder was put into the trough mixer, followed by addition of 95% ethanol, and the mixture was put into the YK-160A swing granulator. (6) The stainless steel plate with particles was incubated in the hot air circulating drying oven (about 80°C) for about 7 h, until the water content was less than 6%.

### 2.2. Analysis of Components of Huzhang Tongfeng Granule Based on LC-MS/MS

Briefly, 12 g of Huzhang Tongfeng granule particles was weighed, followed by addition of 50 mL hot water. The mixture underwent ultrasonication for 10 min, and then 200 *μ*L extract was filtrated with 0.45 *μ*m microporous membrane and detected with UHPLC (SHIMADZU, Kyoto, Japan). The analytical instrument of this experiment was Triple TOF5600+, AB SCIEX instrument. The ion source was ESI. Chromatographic conditions were set as follows: chromatographic column: SHIMADZU InertSustain C18 (100 × 2.1 mm, 2 *μ*m); column temperature: 35°C; velocity of flow: 0.30 mL/min; mobile phase: A. Equate = “acetonitrile,” B. Equate = “0.1% CH_3_COOH-H_2_O”; and elution gradient: 0 min A: 0% B: 100%, 10 min A: 50% B: 50%, 13 min A: 95% B: 5%, 14 min A: 0% B: 100%, 15 min A: 0% B: 100%. Mass spectrometry conditions were set as follows: the scanning range was 100–1,500 *m*/*z*; scanning mode: DIA; capillary voltage: 5,000 V (positive) and 4,500 V (negative); and capillary temperature: 500°C, DP: 60 V, CE: 35 V, CES: 15 V.

### 2.3. The Establishment of the Lentiviral Vector Carrying Cyr61 shRNA

The lentiviral vector expressing green fluorescent protein (GFP) and rat Cyr61 shRNA was constructed by GeneChem Co., Ltd. (Shanghai, China). An shRNA sequence targeting the rat Cyr61 sequence (GenBank NM_031327) was designed as follows: 5′-GAGGAATGGGTCTGTGATGAA-3′ (Cyr61). The lentivirus-GFP (LV-GFP) expressing only GFP was used as a blank control. The randomly selected nonsense sequence 5′-TTCTCCGAACGTGTCACGT-3′ (Cyr61) was used as an additional negative control. The recombinant lentiviruses encoding Cyr61 shRNA and negative controls (LV-GFP and LV-Cyr61) were prepared for a titer of 2*E*^+9^ transfection units (TU)/mL.

### 2.4. Animals

Male Sprague-Dawley (SD) rats weighing 250 ± 20 g were purchased from the Shanghai Laboratory Animals Center (SLAC) Co., Ltd. (Certificate No. 2015000552939). Animals were maintained under specific pathogen-free conditions. All experiments were performed according to the guidelines of the Committee on Protection, Welfare and Ethics of Experimental Animals in Yueyang Hospital of Integrated Traditional Chinese and Western Medicine affiliated to Shanghai University of Traditional Chinese Medicine (No. 18816).

### 2.5. Experimental Model of Acute Gout (MSU-Induced Inflammation)

An acute GA model was induced by a technique described by Coderre and Wall [21] with minor modifications. Briefly, rats were anesthetized with 1% pentobarbital sodium (40 mg/kg, i.p.), the syringe was inserted into the ankle joint cavity at an angle of 45° to the tibia from the lateral side of the right ankle joint, and 0.2 mL of MSU (Sigma-Aldrich, St. Louis, MO, USA) suspension (2.5 g/100 mL) was injected. In the normal control group, the right ankle joint was injected with the same amount of normal saline. Contralateral bulging of the joint capsule was considered as a successful injection. The model was made at 1 h before intragastric administration on the 5th day.

### 2.6. Animal Grouping and Administration Method

After adaptive feeding for 1 week, 20 SD rats were randomly and evenly divided into four groups as follows: normal control group, gout model group, and Huzhang groups (low-dose and high-dose). The adult dose of Huzhang Tongfeng granule was 24 g per day according to the adult standard weight of 70 kg, the rat coefficient was 6.25, and the daily dose of rats was 2.14 g/kg. According to the daily weight change of rats, the dosage of low- and high-dose Huzhang groups was 2.14 g/kg and 8.56 g/kg, respectively. An equal volume of distilled water was administered by gavage in the normal control and model groups. The intragastric volume was calculated by 1 mL/100 g once a day for 7 days.

In the second part of the experiment, 25 SD rats were randomly and evenly divided into five groups as follows: normal control group, gout model group, Huzhang group (high-dose), Huzhang + shRNA group, and colchicine group. In the Huzhang + shRNA group, the method of intragastric administration was the same as that in the Huzhang group, and rats received injections (2 *μ*L lentivirus solution, titer 1*E*^+7^ TU/mL) into the articular cavity immediately after the model was established by MSU administration, and the microinjection of lentiviral vectors was performed as previously described [22, 23]. The dosage of colchicine group was 1 × 10^−4^ g/kg before modeling and 3 × 10^−4^ g/kg after modeling. The methods of intragastric administration in the other groups were the same as before.

As a classical drug for the treatment of acute gout, colchicine can inhibit the chemotaxis, adhesion, and phagocytosis of neutrophils, reduce the release of prostaglandins and leukotrienes by monocytes and neutrophils, and suppress the production of IL-6 [24]. Therefore, colchicine was selected as the positive control drug in this study.

### 2.7. Joint Swelling Evaluation

Previous study has shown [25] that joint swelling is a tool for evaluating gout inflammation. Kawasaki Mitutoyo (Mitutoyo, Kawasaki, Japan) digital caliper was used to determine joint swelling with a minimum accuracy of 0.01 mm [26]. The inflammation of the joint was the ratio of the circumference of 0.5 mm under the ankle joint of the right hind foot to that of the normal control, and the value of more than 1.10 was classified as inflammation.

### 2.8. H&E Staining of Synovium

The rat was anesthetized at 72 h after MSU injection, and synovium from right ankle joint was collected. The collected synovium was fixed with 10% neutral formalin solution, decalcified with 5% nitric acid, routinely dehydrated, sliced, and finally stained with hematoxylin-eosin (H&E), and the local histopathological changes were observed under the light microscope.

### 2.9. Immunohistochemistry of Synovium

The expressions of Cyr61, IL-1*β*, TNF-*α*, and IL-6 in synovium were determined by immunohistochemistry. The synovium was placed in liquid formalin and embedded in paraffin. The tissue embedded in formalin was sliced into sections with a thickness of 6–8 *μ*m. The slices were washed with distilled water and phosphate-buffered saline (PBS) (Sigma-Aldrich, St. Louis, MO, USA) for dewaxing. Then, the slices were repaired by microwave in ethylenediaminetetraacetic acid (EDTA) (Solarbio, Beijing, China) buffer. The endogenous peroxidase was blocked by a mixture of formalin and 3% hydrogen peroxide for 15 min. The sections were washed with PBS for three times, and the non-specific antigen site was blocked with fetal bovine serum for 30 min. Subsequently, the sections were incubated with the primary antibody at 37°C for 1 h and then washed with PBS for three times, followed by incubation with secondary antibody at 37°C for 30 min. The sections were developed with diaminobenzidine (DAB) (Sigma-Aldrich, St. Louis, MO, USA) and stained with hematoxylin and applied to form neutral sesame oil. Images were acquired using a light microscope (Olympus, Tokyo, Japan) and analyzed with the image processing software (Image-Pro Plus 6.0).

The following antibodies were used in the present study: mouse anti-CCN1/Cyr61 (1 : 200 diluted, Abcam, Cambridge, MA, USA), rabbit anti-IL-1*β*, rabbit anti-TNF-*α*, and mouse anti-IL-6 (1 : 150 diluted, all from BioTNT, Shanghai, China) and the secondary antibodies goat anti-mouse IgG HRP and goat anti-rabbit IgG HRP (1 : 2,500 diluted, BioTNT, Shanghai, China).

### 2.10. Preparation of Drug-Containing Serum

After 1 week of adaptive feeding, 14 SD rats were randomly and evenly divided into two groups as follows: blank group and Huzhang group. The rats in the Huzhang group were perfused with an aqueous solution of Huzhang Tongfeng granule. According to the principle of serum pharmacology of TCM and previous research [27], each rat was given a dose of 3.424 g/kg (10 times as much as the normal dose of a human). The blank group was given distilled water of the same volume. The intragastric volume was calculated by 1 mL/100 g, twice a day for 5 days. The blood was taken through the abdominal aorta at 2 h after the last administration, and the drug-containing serum was separated by centrifugation at 4°C for 1 h. The drug-containing serum was inactivated in a water bath, filtered, and sterilized. The Dulbecco's Modified Eagle Medium (DMEM) (HyClone, Logan, UT, USA) supplemented with drug-containing serum and DMEM containing 10% blank serum were prepared.

### 2.11. Synovial Cell Culture and Administration Method

Synovial tissue specimens were obtained from rat ankle joints. Synovial cells (mainly fibroblast-like synoviocytes (FLSs)) were isolated from tissue explants and cultured as previously described [28]. Briefly, each sample was cut into small pieces and placed into a digestion solution containing collagenase (type I) (Sigma-Aldrich, St. Louis, MO, USA) for 1-2 h with brief mixing. After centrifugation, EDTA was added to the precipitate for digestion for 20−30 min, and the digestion was terminated when single- or dispersed-cell masses were observed under the microscope. Then, the cells were collected and cultured in DMEM supplemented with 10% fetal bovine serum (FBS) (Sigma-Aldrich, St. Louis, MO, USA) and 1% streptomycin and penicillin (Solarbio, Beijing, China) at 37°C with 5% CO_2_. After overnight incubation, nonadherent cells were removed, and adherent cells were seeded into 6-well culture plates at a density of 3 × 10^5^ cells/mL. All experiments were performed with cells obtained after passage 3.

In the blank group, 200 *μ*L of blank rat serum was added. In the model group, 200 *μ*L of MSU suspension and 200 *μ*L of blank rat serum were added. In the Huzhang group, 200 *μ*L of MSU suspension and 200 *μ*L of rat serum of the Huzhang group were added. In the Huzhang + shRNA group, a diluted lentivirus solution (titer was 1*E*^+7^) was added on the basis of the Huzhang group. All groups were supplemented with DMEM to a total volume 2 mL and cultured at 37°C with 5% CO_2_ for 48 h. FLSs and supernatants were harvested and frozen at −80°C.

### 2.12. RT-PCR

Total RNA was extracted from tissues or cells using TRIzol reagent (Invitrogen, Carlsbad, CA, USA), and messenger RNA (mRNA) was reversely transcribed into cDNA using cDNA reverse transcription kit (BioTNT, Shanghai, China). RT-PCR was performed using SYBR Green Master Mix (Thermo Fisher, Eugene, OR, USA) according to the manufacturer's instructions. PCR primers (BioTNT, Shanghai, China) used for RT-PCR were shown in Table 2. Data were collected, and quantitative analysis was performed using an ABI Prism 7300 sequence detection system (Applied Biosystems, Foster City, CA, USA). Glyceraldehyde-3-phosphate dehydrogenase (GAPDH) was chosen as the housekeeping gene, and the relative expressions of target genes were calculated by the 2^−ΔCt^ method.

### 2.13. Western Blotting Analysis

Equal amounts of whole joint tissue were lysed in lysis buffer, and tissue proteins were extracted. The cells were lysed, and the supernatant was used for protein quantification. GAPDH was used as a protein loading control. The following primary antibodies were used in the subsequent experiment: rabbit anti-IL-1*β*, rabbit anti-TNF-*α*, mouse anti-IL-6 (all from Abcam, Cambridge, MA, USA), rabbit polyclonal anti-Cyr61 (Invitrogen, Carlsbad, CA, USA), and rabbit anti-GAPDH (Cell Signaling Technologies, Beverly, MA, USA). The secondary antibodies used were goat anti-rabbit IgG HRP, goat anti-mouse IgG HRP, and donkey anti-goat IgG HRP (Beyotime Biotechnology, Shanghai, China). The target proteins were examined using a Tanon-5200 imaging system (Tanon Science and Technology Corporation, Shanghai, China) and visualized with autoradiography film.

### 2.14. ELISA

The levels of IL-1*β*, TNF-*α*, and IL-6 in serum or cell supernatant were determined using ELISA (sensitivity 0.8–300 pg/mL; R&D Systems, Minneapolis, MN, USA; ELISA kits were from BioTNT, Shanghai, China) according to the manufacturer's instructions.

### 2.15. Statistical Analysis

Data were analyzed with GraphPad Prism 7.0 software (GraphPad Software, La Jolla, CA, USA) and expressed as mean ± SEM or median (range). Multiple comparisons among groups were performed by one-way ANOVA. Comparisons in groups were performed by Tukey's test. Repeatedly measured data were analyzed by repeated measurement analysis of variance. Data with nonnormal distributions were analyzed by nonparametric test. *P* values <0.05 were considered as statistically significant.

## 3. Results

### 3.1. Determination of Effective Components in Huzhang Tongfeng Granule by LC-MS/MS

The raw data of LC-MS were imported into MS-DIAL 3.70 software for preprocessing. The extracted peak information was compared with the database, and the three databases of MassBank, Respect, and GNPS (a total of 14,951 records) were searched in the whole database.  Table 3 shows the qualification list.

### 3.2. Huzhang Tongfeng Granule Has an Obvious Inhibitory Effect on the Rat Model of Acute GA in a Dose-Dependent Manner

**Figure 1:**
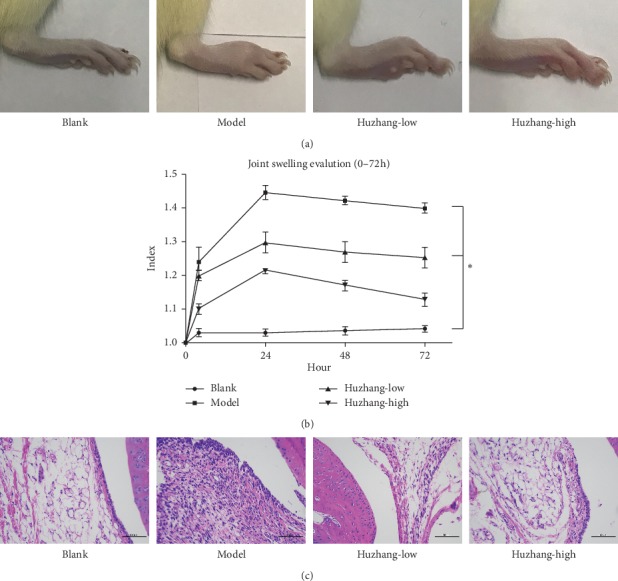
(a) The pictures of arthritis from the blank, model, Huzhang-low, and Huzhang-high groups after 72 h. (b) The ratio of the right foot to the left foot (normal control) from 0 h to 72 h. (c) H&E staining of synovial tissue from each group (200x). ^*∗*^*P* < 0.05 vs. blank.

After modeling, the right ankle joint of rats was obviously swollen with red and hot manifestations, and some even lifted the swollen joint off the ground. After the establishment of the model, the degree of swelling was gradually increased with time, especially in the model group, while the change of swelling degree was slow and recovered quickly in the normal control group (Figure 1(a)). After 72 h of modeling, the degree of joint swelling in the high-dose Huzhang group was significantly lower compared with the model group, while there was no significant improvement in the low-dose group (Figure 1(b)). Histological observation showed that the synovial tissue structure of the normal control group was clear, the synovial cells were arranged in a single layer, the cell morphology was normal, and there was no inflammatory cell infiltration. In the model group, synovial inflammation was obvious, synovial cells proliferated, and there was more inflammatory cell infiltration. The proliferation of synovial cells was not obvious, and the number of inflammatory cells in the low-dose Huzhang group was less compared with the model group. Synovial cell proliferation and inflammatory cell infiltration were significantly reduced in the high-dose Huzhang group (Figure 1(c)).

### 3.3. Huzhang Tongfeng Granule Reduces the Expressions of Cyr61 and Related Inflammatory Factors

**Figure 2:**
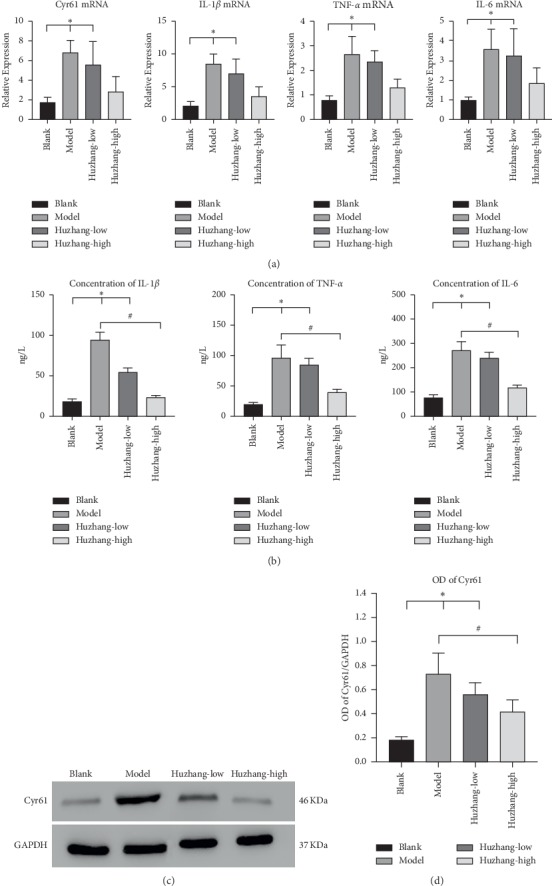
(a) Relative expressions of Cyr61, IL-1*β*, TNF-*α*, and IL-6 at the mRNA level in the blank, model, Huzhang-low, and Huzhang-high groups by PCR. (b) The concentrations of IL-1*β*, TNF-*α*, and IL-6 were verified by ELISA in the same groups. (c) The protein level of Cyr61 was verified by Western blotting analysis in the rat synovial tissue from the same groups. (d) The protein level in different groups was expressed as a ratio to GAPDH. ^*∗*^*P* < 0.05 vs. blank, ^#^*P* < 0.05 vs. model.

**Figure 3:**
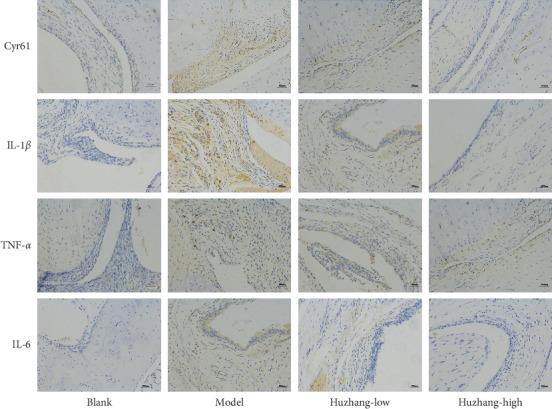
The IHC pictures of arthritis from the blank, model, Huzhang-low, and Huzhang-high groups (200x).

Studies have shown that the inflammatory process of acute GA is closely associated with IL-1*β*, TNF-*α*, and IL-6 [4, 6, 29]. Other studies have demonstrated that Cyr61 participates in the pathogenesis of inflammatory diseases [15]. In our study, we examined the Cyr61 concentration in synovial tissues from MSU-induced rat models. The results showed that the Cyr61 expression at both the protein and mRNA levels was increased (*P* < 0.05) in MSU-induced rat models, and the expression of Cyr61 was significantly decreased in the high-dose Huzhang group (Figures 2(a), 2(c), and 2(d)). The expressions of IL-1*β*, TNF-*α*, and IL-6 at the mRNA and protein levels in the high-dose Huzhang group were significantly lower compared with the model group (Figures 2(a) and 2(b)), which was consistent with the immunohistochemical findings of joint tissues (Figure 3).

The results of immunohistochemistry showed that there was almost no expression of Cyr61 in synovial tissue of the normal control group, while the expression of Cyr61 was increased in synovial tissue of the model group and decreased in the high-dose Huzhang group, which was consistent with the inhibitory effect on inflammatory factors (Figure 3).

### 3.4. The Anti-Inflammatory Ability of Huzhang Tongfeng Granule Is Decreased after the Expression of Cyr61 Is Blocked

**Figure 4:**
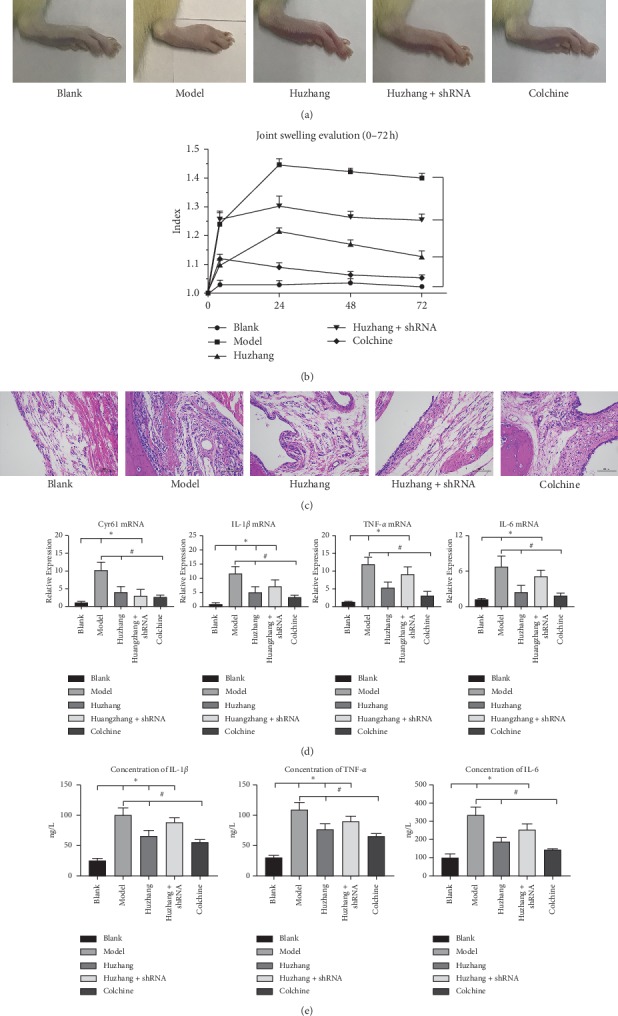
(a) The pictures of arthritis from the blank, model, Huzhang, colchicines, and shRNA groups after 72 h. Colchicine was used as a positive control. (b) The ratio of the right foot to the left foot (normal control) from 0 h to 72 h. (c) H&E staining of synovial tissue from each group (200x). (d) Relative expressions of Cyr61, IL-1*β*, TNF-*α*, and IL-6 at the mRNA level in the same group by PCR. (e) The concentrations of IL-1*β*, TNF-*α*, and IL-6 were verified by ELISA in the same groups. ^*∗*^*P* < 0.05 vs. blank, ^#^*P* < 0.05 vs. model.

In the second part of the experiment, we used a specific Cyr61 interference lentivirus to deplete the expression of Cyr61 in synovial tissue in the Huzhang + shRNA group. The joint swelling of rats in the Huzhang group and colchicine group was significantly alleviated, while there was no significant improvement in the Huzhang + shRNA group compared with the model group (Figures 4(a)–4(c)). The expression of Cyr61 at the mRNA level in the Huzhang group and colchicine group was markedly decreased, and the expressions of IL-1*β*, TNF-*α*, and IL-6 were also significantly decreased. In the Huzhang + shRNA group, the expressions of IL-1*β*, TNF-*α*, and IL-6 at the mRNA and protein levels were not significantly different from those of the model group (Figures 4(d) and 4(e)).

### 3.5. Huzhang Tongfeng Granule Inhibits the Inflammation of MSU-Stimulated FLSs Partly by Inhibiting the Expression of Cyr61

**Figure 5:**
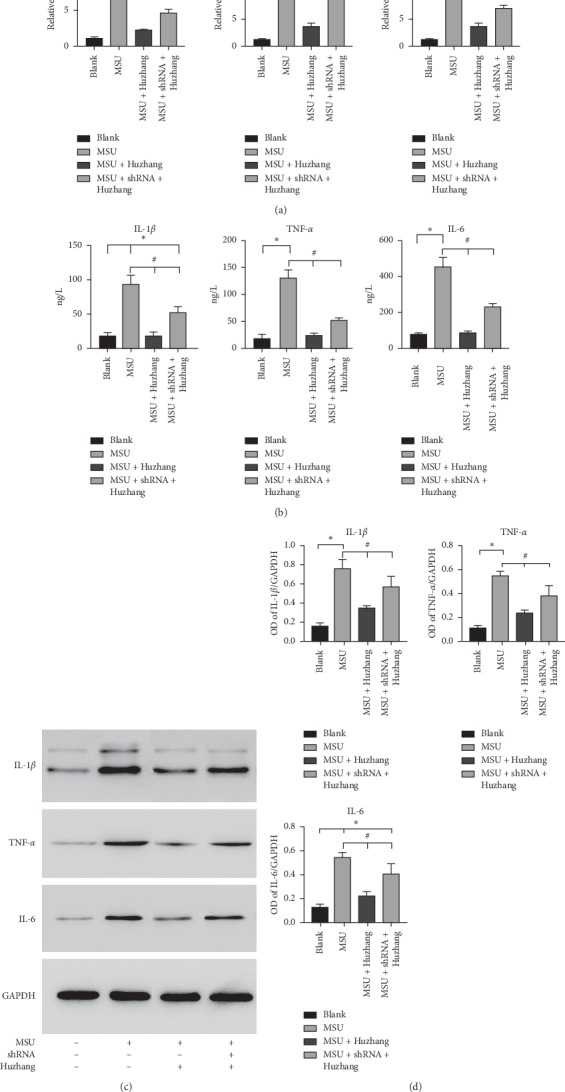
(a) Relative expressions of IL-1*β*, TNF-*α*, and IL-6 at the mRNA level in the blank, MSU-induced, and Huzhang groups with or without shRNA interference. (b) Protein levels of IL-1*β*, TNF-*α*, and IL-6 from the same groups by ELISA. (c) Protein levels of IL-1*β*, TNF-*α*, and IL-6 in the same groups. (d) The protein level in the same groups was expressed as a ratio to GAPDH. ^*∗*^*P* < 0.05 vs. blank, ^#^*P* < 0.05 vs. model.

We analyzed the effect of drug-containing serum of Huzhang Tongfeng granule on the expressions of IL-1*β*, TNF-*α*, and IL-6 in MSU-stimulated FLSs. The results showed that the expressions of IL-1*β*, TNF-*α*, and IL-6 in FLSs induced by MSU were decreased by the addition of serum-containing Huzhang Tongfeng granule. After depletion of Cyr61, the expressions of IL-1*β*, TNF-*α*, and IL-6 both at the protein and mRNA levels in the serum group containing Huzhang Tongfeng granule were significantly decreased, while the effect was weaker compared with the Huzhang Tongfeng granule containing serum group (Figures 5(a)–5(d)). These results suggested that Huzhang Tongfeng granule could partly inhibit the secretion of IL-1*β*, TNF-*α*, and IL-6 by downregulating the expression of Cyr61.

## 4. Discussion

Huzhang Tongfeng granule is one of the most efficient TCMs in the treatment of acute GA. Our previous study has confirmed that Huzhang Tongfeng granule antagonizes acute gout by inhibiting neutrophil activation, chemotaxis, and aggregation to inflammatory sites and reducing the release of inflammatory mediators. However, the regulatory mechanism of inflammatory factors remains unclear.

The acute attack of gout is an acute inflammatory process induced by MSU crystals. It begins with the interaction between urate crystals and resident mononuclear phagocytes and induces a series of inflammatory reactions with the participation of phagocytes and neutrophils [30–32]. Immune system disorder; infiltration of monocytes, macrophages, and neutrophils; activation of mast cells; and production of a large number of cytokines are all important links in the mechanism of gout inflammation. In the whole process of inflammation, many inflammatory factors, such as IL-1*β*, TNF-*α*, IL-6, and neutrophil chemokine, are involved in the induction and expansion of inflammation [33–35]. At present, we have a certain understanding of the inflammatory factors involved in the inflammatory process of GA. However, the mechanism of each link in the inflammatory process is not completely clear and needs to be further studied.

Cyr61 is related to the pathogenesis of the autoimmune disease [16], and its influence on immune inflammatory diseases has attracted more and more attention. With the further study of Cyr61 in RA, it has been confirmed that Cyr61 plays a clear role in joint inflammation and bone destruction [11–13]. As a type of inflammatory factor, whether Cyr61 also participates in and plays an important role in the inflammatory response of gout is worth studying.

In vivo experiment revealed that the expressions of IL-1*β*, TNF-*α*, and IL-6 were significantly upregulated in synovium and serum of MSU-induced rat models, which was consistent with the characteristics of GA inflammatory reaction. We also found that Cyr61 was highly expressed in synovium. Histological observation showed that Huzhang Tongfeng granule could reduce the local inflammatory reaction and local inflammatory cell infiltration in MSU-induced rat models. Huzhang Tongfeng granule could downregulate the expressions of IL-1*β*, TNF-*α*, and IL-6 in a dose-dependent manner. In order to confirm the anti-inflammatory effect of Huzhang Tongfeng granule by downregulating Cyr61, we then used a specific Cyr61 interference lentivirus to deplete the Cyr61 expression in synovium, and the results showed that the ability of Huzhang Tongfeng granule to inhibit the expressions of inflammatory factors was decreased.

In vitro, MSU suspension was used to stimulate rat FLS. It was found that MSU could induce FLSs to secrete inflammatory factors, IL-1*β*, TNF-*α*, and IL-6. The expressions of IL-1*β*, TNF-*α*, and IL-6 in FLSs stimulated by MSU were significantly decreased after intervention with drug-containing serum of Huzhang Tongfeng granule. When the expression of Cyr61 was depleted, the ability of Huzhang Tongfeng granule to inhibit inflammatory factors was decreased, which was consistent with the experimental results in vivo.

Our study found that Cyr61 was highly expressed in synovial tissues from MSU-induced rat models. Huzhang Tongfeng granule could downregulate the expressions of Cyr61, IL-1*β*, TNF-*α*, and IL-6. When the expression of Cyr61 was inhibited, the ability of Huzhang Tongfeng granule to inhibit these inflammatory factors was also weakened, suggesting that Huzhang Tongfeng granule could inhibit the inflammatory response of acute gout by downregulating the expression of Cyr61. Therefore, the molecular biology mechanism for Huzhang Tongfeng granule in treating acute GA could be attributed to the reduction of high expression of Cyr61.

## 5. Conclusions

In the present study, we showed that Huzhang Tongfeng granule could reduce the inflammatory response induced by MSU crystal, to some extent, by inhibiting the expression of Cyr61.

## Figures and Tables

**Table 1:** Ingredients of Huzhang Tongfeng granule.

Latin scientific name	Plant part (s)	Amount (g)
Polygonum cuspidate Sieb. et Zucc.	Radix and rhizoma	11.0
Artemisia scoparia Waldst. et Kit. or artemisia capillaris Thunb.	Stem and foliage	11.0
Notopterygium incanum Ting ex H. T. Chang	Radix and rhizoma	3.74
Angelica pubescens Maxim. f. biserrata Shan et Yuan	Radix	3.74
Saposhnikovia divaricata (Trucz.) Schischk.	Radix	6.25
Stephania tetrandra S. Moore	Radix	4.5
Angelica sinensis (Oliv.) Diels	Radix	7.5
Pueraria lobata	Radix	7.5
Polyporus umbellatus (Pers.) Fries	Sclerotium	7.5
Atractylodes lancea (Thunb.) DC. or Atractylodes chinensis (DC.) Koidz.	Rhizoma	3.74
Pinus tabulac formis Carr. or Pinus massoniana Lamb.	Berous or branching nodes	5.0
Glycyrrhiza uralensis Fisch.	Radix and rhizoma	4.5

**Table 2:** Specific primers used in RT-PCR.

Name	Primer	Sequence (5′-3′)
Cyr61	FW	AAAGGTCTCCTGGGTTTC
	RV	ACTGCGTTACTGTCCATC

IL-1*β*	FW	TGTGATGTTCCCATTAGAC
	RV	TCTTTGGGTATTGTTTGG

TNF-*α*	FW	CCACGCTCTTCTGTCTACTG
	RV	GCTACGGGCTTGTCACTC

IL-6	FW	GTTGCCTTCTTGGGACTG
	RV	ACTGGTCTGTTGTGGGTG

GAPDH	FW	GGAGTCTACTGGCGTCTTCAC
	RV	ATGAGCCCTTCCACGATGC

**Table 3:** Up to 20 chemical constituents identified in Huzhang Tongfeng granule.

Peak number	Formula	Identification
1	C₂₁H₂₄N₂O₂	Catharanthine
2	C_6_H_14_N_4_O_2_	Arginine
3	C₁₅H₁₀O₄	Daidzein
4	C_21_H_21_C_l_O_10_	Pelargonidin
5	C_15_H_10_O_4_	Flavonol
6	C_15_H_10_O_5_	Apigenin
7	C_15_H_10_O_6_	Kaempferol
8	C_15_H_10_O_7_	Quercetin
9	C_7_H_12_O_6_	Quinic acid
10	C_4_H_6_O_5_	Malic acid
11	C_15_H_10_O_5_	Emodin
12	C_6_H_14_O_6_	Mannitol
13	C_22_H_22_O_9_	Ononin
14	C_16_H_22_O_10_	Swertiamarin
15	C_20_H_24_O_9_	Ginkgolide A
16	C_15_H_12_O_5_	Isoliquiritigenin
17	C_42_H_62_O_16_	Glycyrrhizin
18	C_18_H_16_O_8_	Rosmarinic acid
19	C_5_H_9_NO_2_	Proline
20	C_16_H_12_O_7_	Isorhamnetin

## Data Availability

All the data used to support the findings of this study were supplied by Dr. Mi Zhou under license and so cannot be made freely available. Requests for access to these data should be addressed to Dr. Mi Zhou (e-mail:vieky2866@163.com).
